# Pre-Harvest Non-Typhoidal *Salmonella* Control Strategies in Commercial Layer Chickens

**DOI:** 10.3390/ani14243578

**Published:** 2024-12-11

**Authors:** Roshen N. Neelawala, Lekshmi K. Edison, Subhashinie Kariyawasam

**Affiliations:** Department of Comparative, Diagnostic, and Population Medicine, College of Veterinary Medicine, University of Florida, Gainesville, FL 32608, USA; roshen.neelawala@ufl.edu (R.N.N.); edison.le@ufl.edu (L.K.E.)

**Keywords:** antimicrobial resistance, biosecurity, vaccination, feed additives, food safety, layer chickens, non-typhoidal *Salmonella*, pre-harvest control, poultry health

## Abstract

Non-typhoidal *Salmonella* (NTS) infections in poultry, particularly in commercial-layer chickens, pose a critical risk to food safety and public health worldwide. NTS bacteria can remain undetected in poultry flocks, contaminating products and potentially leading to gastroenteritis in humans. This review examines pre-harvest control strategies for NTS in layer chickens, including biosecurity protocols, vaccinations, feed additives, genetic selection, and environmental management. These strategies have substantially reduced *Salmonella* colonization and product contamination rates in the commercial layer industry. By evaluating these strategies, this review highlights the importance of integrated control measures to limit NTS colonization, reduce antimicrobial resistance, and improve poultry health. This review aims to provide producers, researchers, and policymakers with insights into effective practices to minimize *Salmonella* contamination and enhance both animal and human health outcomes.

## Simple Summary

*Salmonella* poses a significant threat to poultry farming, as it can asymptomatically infect flocks, ultimately leading to foodborne illnesses when contaminated poultry products reach consumers. This review focuses on various strategies for controlling *Salmonella* colonization in commercial layer chickens at the pre-harvest level. The approaches discussed include enhancing farm biosecurity, using vaccines, incorporating feed additives, and improving genetic resistance to infection through selective breeding. Each of these approaches offers unique benefits, such as preventing the introduction of pathogens, enhancing immunity against *Salmonella*, and/or improving intestinal health to reduce *Salmonella* colonization. Therefore, a combined approach is essential for minimizing the risk of *Salmonella*, promoting food safety, and supporting sustainable poultry production.

## 1. Introduction

Non-typhoidal *Salmonella* (NTS) refers to a group of *Salmonella* serovars associated with gastroenteritis in humans. Notable serovars include *S.* Enteritidis, *S.* Typhimurium, *S.* Kentucky, *S.* Newport, *S.* Javiana, *S.* Heidelberg, *S.* Muenchen, *S.* Infantis, *S.* Braenderup, *S.* Saintpaul, *S.* Montevideo, and *S.* Thompson [1]. Some of these serovars pose a significant threat due to their zoonotic transmission from poultry to humans [2]. Poultry products, such as meat and eggs, serve as critical reservoirs of NTS; therefore, they contribute significantly to foodborne disease outbreaks globally [3]. Among NTS serovars, *S.* Enteritidis and *S.* Typhimurium are the most commonly linked to foodborne illnesses worldwide, particularly in the United States and the European Union, stemming from the consumption of eggs and egg products [1,4]. In commercial poultry operations, especially in layer chickens, NTS colonization usually occurs without symptoms, allowing *Salmonella* to persist undetected in flocks [5]. This silent carriage has a direct impact on public health by facilitating contamination of poultry environment and poultry products. Therefore, controlling NTS in poultry has become an urgent global priority to reduce foodborne illnesses and underscores the need for effective pre-harvest interventions. The occurrence and spread of NTS in commercial layer chicken facilities can vary widely depending on the geographical region, farm management practices, and the effectiveness of biosecurity measures [6]. In the United States, *Salmonella* is the leading cause of foodborne infections, and poultry products have consistently been identified as major contributors [5].

**Figure 1:**
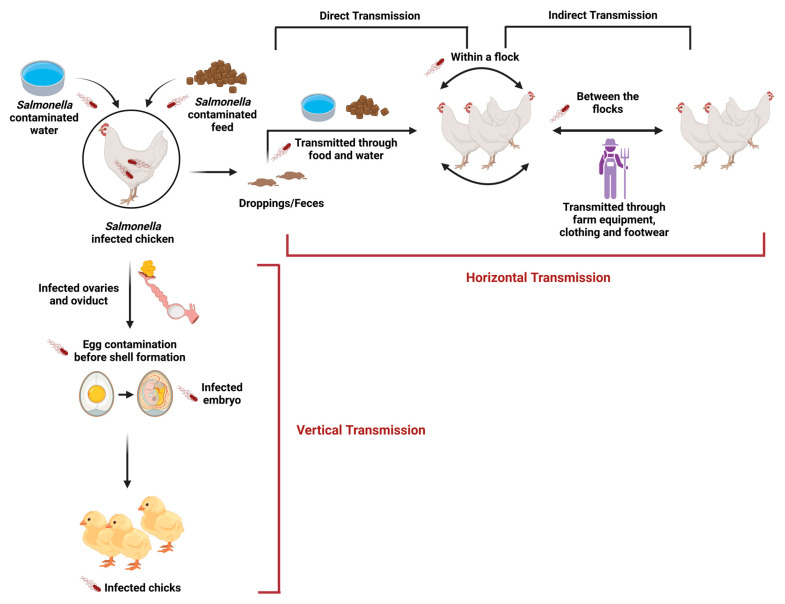
Transmission pathways of *Salmonella* in poultry (created with BioRender.com).

In avian hosts, NTS colonization of the gastrointestinal tract (GIT) is a complex process involving the adhesion of bacteria to intestinal epithelial cells and subsequent invasion. During this course of colonization, the NTS evolved several adaptive mechanisms to evade the host immune system. These survival mechanisms include modulation of the local immune environment and the use of virulence factors that enable the pathogen to exist within the host without causing overt clinical manifestations [7,8,9]. In poultry, the transmission of NTS can occur through vertical or horizontal (Figure 1) pathways, necessitating that the pre-harvest control strategies focus on various farm management aspects. Vertical transmission occurs when *Salmonella* is transmitted from the breeder hens to their progeny through contaminated eggs. This is observed when *Salmonella* contaminates the hen’s reproductive organs, particularly the ovaries or oviducts, leading to egg contamination before shell formation. Such infected eggs can hatch into chicks carrying the pathogen throughout their development, thereby perpetuating the infection cycle. Horizontal transmission of NTS implies pathogen dissemination within a flock or between different flocks, often through direct or indirect contact. The fecal-oral route is the main route of infection; wherein other birds ingest *Salmonella* from contaminated droppings by pecking contaminated litter or drinking water. Poor quality feed or water sources may act as reservoirs, bringing the pathogen into contact with healthy birds. Other possible vectors include contaminated farm equipment, clothing, rodents, and footwear from farm workers [8].

From a production point of view, NTS can lead to economic losses in commercial layer operations by decreasing egg production, increasing mortality in severe cases, and raising the cost of implementing control measures [8]. Although many NTS infections in poultry are subclinical, their presence may still subtly affect the health status, impacting feed conversion efficiency, growth rate, and overall productivity of the flock [10]. From a public health perspective, NTS has a significant impact as a leading cause of foodborne illness worldwide. Contaminated poultry products, particularly eggs, are recognized as a main source of NTS to humans. Human infections generally lead to self-limiting gastroenteritis, which presents typical symptoms of diarrhea, abdominal pain, fever, and vomiting [9]. However, in the elderly, children, and immunocompromised individuals, it can lead to serious extraintestinal infections. The ability of *Salmonella* to acquire antimicrobial resistance presents an additional challenge to veterinary and human health care [11].

Controlling NTS during the pre-harvest stage in commercial-egg production is critical in reducing foodborne diseases due to *Salmonella*, limiting the spread of antimicrobial resistance, and improving public confidence in food safety [12]. Therefore, this review comprehensively evaluates various pre-harvest control strategies that benefit poultry producers, researchers, and policymakers in effectively implementing NTS control measures.

## 2. Risk Factors Influencing *Salmonella* Contamination in the Pre-Harvest Stage

Understanding the factors influencing *Salmonella* contamination in the pre-harvest stage is essential for developing effective control strategies in commercial layer chicken operations. Various environmental, management, biological, and microbial factors contribute to the risk of *Salmonella* presence in poultry flocks, each playing a unique role in facilitating the spread and persistence of these bacteria [13,14].

### 2.1. Environmental Factors

Environmental conditions within the poultry housing system significantly influence *Salmonella* contamination [15]. Temperature, humidity, and ventilation all impact *Salmonella* survival and transmission within poultry facilities. Higher temperatures and humidity can increase bacterial survival rates in litter, dust, feces, and surfaces, creating an environment conducive to pathogen persistence [16]. Ventilation in poultry houses significantly influences *Salmonella* contamination levels, as large volumes of air can transport bacteria from litter and dust into the aerosol environment within the sheds. Mechanical ventilation systems, such as tunnel ventilation, move substantial air volumes through poultry sheds, potentially aerosolizing *Salmonella* and enabling its dispersal inside and outside the housing unit. However, external levels are usually lower than internal *Salmonella* concentrations [17]. Nonetheless, inadequate light and ventilation can increase stress among birds and weaken their immune responses, making them more susceptible to infections like *Salmonella* [18,19,20]. Additionally, the type and quality of litter used in non-cage housing systems play a role; soiled or moist litter can support *Salmonella* proliferation, especially if it is not changed or managed regularly [21,22]. Maintaining optimal environmental conditions through temperature control, adequate ventilation, and regular litter management is crucial to minimizing contamination risks.

### 2.2. Farm Management Practices

Poor hygiene practices, such as inadequate cleaning and disinfection of poultry houses, equipment, and feed areas, create environments where *Salmonella* can thrive and spread. Farm biosecurity practices are also essential, as inadequate restrictions on personnel movement, ineffective entry points, and equipment disinfection can introduce or spread *Salmonella* within flocks. Farm management includes implementing proper waste disposal systems and rigorous cleaning schedules [23,24]. Additionally, feed and water management play a role; contaminated feed and water are primary sources of *Salmonella*, and failure to routinely monitor and sanitize these inputs increases the risk of introducing the pathogen to chickens [25]. Ensuring regular inspections, enforcing strict biosecurity protocols, and implementing cleaning schedules help mitigate the risk of contamination [23,24].

### 2.3. Flock Density and Housing Systems

The housing system and flock density directly affect *Salmonella* transmission among birds. In high-density rearing systems, birds are in closer contact, which can facilitate the rapid spread of *Salmonella* through direct and indirect contact, including via droppings, feathers, and feed. Cage-free and free-range systems, while offering benefits for animal welfare, may pose unique challenges for *Salmonella* control [26]. In free-range systems, birds may be exposed to contaminated soil, insects, or wild animals, all of which can serve as reservoirs for *Salmonella*. In addition, free-range environments make it difficult to control environmental contamination sources, adding complexity to management practices [26,27]. However, cage production systems pose a greater risk of *Salmonella* contamination compared to non-cage production systems, such as barn and free-range systems. This increased risk is attributed to several factors inherent to cage systems. Cages usually hold a large number of birds in a confined space, which facilitates the rapid spread of microorganisms, including *Salmonella*. Moreover, the compact environment can cause stress to the hens, weakening their immune system and making them more vulnerable to disease. In addition, the structural layout of cage systems makes effective cleaning and disinfection challenging, allowing *Salmonella* to persist in the environment and further increasing the risk of contamination [15,28,29,30,31]. The recovery rate of *Salmonella* from the dust and fecal samples in caged systems is typically higher than in non-cage systems [32]. Additionally, *Salmonella* persists longer in houses with a deep pit (step-cage houses and cages with a scraper manure disposal system) than in non-cage systems [31]. Controlling flock density, regularly rotating pastures for free-range birds, and maintaining clean housing environments can help minimize the risk of contamination in various housing systems.

### 2.4. Bird Age and Immunity

The age and immune status of birds influence their susceptibility to *Salmonella* infection. Younger chickens with immature immune systems are generally more susceptible to *Salmonella* and are often the focus of preventative strategies such as vaccination. Due to overcrowding, poor nutrition, or suboptimal environmental conditions, birds under stress may experience immune suppression, making them more vulnerable to infections [18,19,20]. Vaccinating chickens against *Salmonella* at an early age has proven to effectively reduce susceptibility by providing them with specific immunity to certain *Salmonella* serovars [33]. Additionally, selective breeding for resistance to pathogens, including *Salmonella*, is an emerging area of interest. Recent studies have identified genetic variations and specific loci associated with immune and disease-resistance traits, indicating the potential for the use of breeding programs to enhance resistance to infections [34]. Ensuring optimal health, nutritional support, and targeted vaccination schedules can strengthen flock immunity, reducing the likelihood of *Salmonella* establishment and transmission.

### 2.5. Feed and Water Quality

Contaminated feed and water are among the primary sources of *Salmonella* entry into poultry flocks [8]. Feed contamination can occur at any stage in the supply chain, including during production, storage, or transport. In particular, animal-based feed ingredients, like fishmeal or meat by-products, have higher contamination risks [35]. Ensuring that feed undergoes processing treatments, such as heat-pelleting, can reduce *Salmonella* presence [36]. Water quality also plays a crucial role in influencing *Salmonella* contamination at the pre-harvest stage, as water systems can serve as reservoirs for *Salmonella* biofilms. This protection allows the bacteria to persist over time within the water systems, contributing to the ongoing risk of contamination in poultry environments. Regular water testing and appropriate water sanitizers can help prevent contamination [37].

### 2.6. Role of Rodents, Wild Birds, and Insects as Vectors

Rodents, wild birds, and insects act as vectors, facilitating the introduction and spread of *Salmonella* in poultry farms. Rodents, in particular, are known carriers of *Salmonella* and can easily transmit the bacteria through droppings, which can contaminate feed, water, and litter [14]. Wild birds that access free-range or cage-free areas can introduce *Salmonella* to poultry through droppings or direct contact. Similarly, flies and other insects can pick up *Salmonella* from contaminated environments and transmit it to birds through feeding or nesting materials [8]. Effective pest control measures are essential in reducing the likelihood of *Salmonella* transmission from these external vectors. Measures such as securing feed storage, installing barriers to restrict wild bird access, and implementing rodent and insect control programs are vital to minimizing contamination risks.

### 2.7. Antimicrobial Resistance (AMR) and Its Impact on Salmonella Control

The rise in antimicrobial resistance (AMR) among *Salmonella* strains is an increasingly concerning topic in pre-harvest control of *Salmonella*. The use of antibiotics in poultry production for growth promotion and disease prevention has contributed to the development of resistant *Salmonella* strains, making infections more challenging to manage [38,39]. Resistant strains can persist longer in the environment and within birds, reducing the efficacy of traditional control measures. AMR complicates treatment options and poses a risk to human health, as resistant *Salmonella* can be transmitted to humans through contaminated poultry products [39]. AMR can also facilitate the horizontal transfer of resistance genes between different bacterial populations within the poultry environment, compounding the issue by increasing the diversity of resistant pathogens present [40]. This prolonged environmental survival and gene transfer capability amplify the risk of resistant *Salmonella* strains spreading to humans, underscoring the critical need to understand AMR’s impact on *Salmonella* dynamics in pre-harvest settings [41,42]

### 2.8. Human Interaction and Farm Personnel Practices

Human interaction, including the activities of farm personnel, is another significant factor influencing *Salmonella* contamination in the pre-harvest stage. Workers can inadvertently introduce *Salmonella* through contaminated clothing, footwear, and equipment if proper biosecurity measures are not followed. Without strict hygiene protocols, personnel moving between different farm areas or flocks can facilitate cross-contamination [43,44]. Producers must commit to training staff on proper hygiene, providing dedicated clothing and equipment for specific zones, and implementing hand-washing and disinfection protocols in order to minimize human-mediated *Salmonella* transmission. Additionally, the establishment of clear visitor policies can reduce the risk of introducing external contamination sources [45].

## 3. Pre-Harvest Control Strategies

### 3.1. Biosecurity Measures

Biosecurity measures are a key component of *Salmonella* control in commercial poultry that have contributed greatly to the current levels of success observed in the industry [46,47,48]. Implementing comprehensive biosecurity measures is fundamental to infection control, as the effectiveness of concurrent preventive interventions is significantly diminished when biosecurity protocols are compromised. Biosecurity protocols encompass diverse preventive measures designed to minimize pathogen introduction and transmission within poultry flocks. These include physical barriers, sanitation protocols, pest management systems, personal protective equipment requirements, and strict personnel movement controls [15,49].

Establishing physical barriers represents a cornerstone of biosecurity protocols in preventing pathogen introduction to poultry facilities. Implementing perimeter fencing and controlled entry points for personnel and vehicles is essential. Enhanced biosecurity measures, such as perimeter fencing and restricted access, can reduce *Salmonella* contamination rates in poultry facilities by up to 80% [50]. Epidemiological studies, also highlighted the effectiveness of stringent biosecurity measures in reducing *Salmonella* contamination in commercial layer farms [14]. Similarly, Samper-Cativiela et al. (2023) also demonstrated the positive impact of physical barriers and entry restrictions in commercial layer facilities in Spain [6]. These studies underscore the importance of well-defined boundaries and access control measures in minimizing *Salmonella* outbreaks [51].

Moreover, the strategic limitation of equipment and vehicle movement between facilities effectively reduces cross-contamination risks. Specific control measures include the implementation of dedicated facility-specific footwear and protective clothing, alongside the adoption of all-in/all-out production systems, which substantially minimizes cross-contamination between different age cohorts [52].

Environmental decontamination protocols targeting water systems, feed storage, and litter management are crucial in preventing *Salmonella* introduction. These protocols aim to substantially reduce microbial load within poultry housing facilities. Research demonstrates that implementing systematic cleaning and decontamination procedures significantly reduces *Salmonella* contamination in broiler housing environments [53]. Surveillance and monitoring are other essential components of an effective biosecurity framework. Regular microbiological testing of flocks, feed supplies, and environmental samples enables the identification of potential contamination sources and informs evidence-based management decisions. These surveillance systems facilitate early detection of *Salmonella* outbreaks, enabling rapid corrective interventions. Integrating biosecurity measures with systematic surveillance enhances the overall efficacy of *Salmonella* control programs in poultry operations [54].

Feed represents a primary vector for pathogen transmission in poultry flocks, with *Salmonella* identified as the predominant biological hazard across animal feed categories [55]. These risks can be mitigated by sourcing ingredients from verified suppliers who maintain rigorous sampling and testing protocols [56]. At the level of the feed mill, the establishment of segregated clean and dirty zones, coupled with strict access controls, significantly reduces the potential for *Salmonella* and other pathogen cross-contamination between raw materials and finished feed products [57]. Another prominent source of *Salmonella* in poultry farms are rodents [14] and insects, and they function as both mechanical and biological vectors in pathogen transmission. The mitigation of these risks requires the implementation of integrated pest management protocols, incorporating physical barriers, strategic trap placement, vegetation management, and systematic chemical intervention through targeted pesticide applications [58].

### 3.2. Vaccination

Although numerous vaccine platforms, such as live attenuated vaccines, inactivated vaccines, subunit vaccines, and emerging options like ghost vaccines, have been tested experimentally for *Salmonella* control in layer operations, only a few of these are currently available for commercial use. The available vaccines include inactivated whole-cell preparations, and live attenuated strains, each type providing distinct benefits in terms of immune response and protection [59]. Over the past few decades, these vaccination efforts have significantly reduced *Salmonella* incidence in poultry flocks over recent decades, contributing to improved food safety outcomes. [60,61]. Studies have demonstrated that vaccination can reduce *Salmonella* prevalence in flocks by as much as 70% [62]. However, vaccine efficacies can vary depending on the *Salmonella* serovar, environmental conditions, and types of poultry.

#### 3.2.1. Live Attenuated Vaccines

Attenuated vaccines against *Salmonella* comprise live bacteria that have been specifically altered to reduce their virulence while preserving essential immunogenic properties needed to trigger a protective immune response in birds. Several live attenuated vaccines are commonly used in commercial poultry production, including commercial layer production, due to their lower cost, ease of manufacture, and simpler administration than inactivated vaccines (Table 1). These vaccines mimic natural infection by adhering to mucosal surfaces and interacting with mucosa-associated lymphoid tissue, thereby eliciting robust humoral and cell-mediated immune responses [63,64,65]. They are also known to provide heterologous protection and confer immunity against serovars beyond the serovar of the specific vaccine strain and protection against a broader range of *Salmonella* compared to killed vaccines [66,67]. Furthermore, live vaccines provide an additional protective mechanism through the competitive exclusion of pathogens, particularly benefiting young chickens with a still-developing gut microflora and an immature immune system [68]. However, live vaccines could persist for extended periods post-vaccination, potentially transferring to poultry products and, subsequently, to consumers [69]. Although these strains are attenuated, their persistence in the environment and poultry products raises concerns for food safety officials and the public. Another downside of this persistence is that these vaccine strains may interfere with pre-harvest *Salmonella* surveillance and performance standards during poultry processing, leading to financial losses for the farmer [59]. To address this issue, novel methods that could discriminate between field strains and vaccine strains of *Salmonella* have been widely used [69].

#### 3.2.2. Inactivated Vaccines

Inactivated vaccines typically consist of whole bacterial cells combined with an adjuvant, where cultured pathogens are rendered non-infectious by inactivation with heat, chemicals, or irradiation before being used for immunization. Inactivated vaccines against *Salmonella* have been extensively studied and utilized in poultry (Table 1) [70,71,72]. These vaccines elicit an immune response without the risk of causing disease, as they contain killed bacteria. In response to the challenges posed by the diversity of *Salmonella* serovars, researchers have developed multivalent inactivated vaccines that demonstrate protective efficacy against an expanded range of serovars, including emerging serovars such as *Salmonella* Infantis [73]. However, inactivated vaccines primarily stimulate humoral immunity rather than mucosal or cell-mediated immunity, which are crucial for controlling intracellular pathogens and may limit the vaccine’s ability to control *Salmonella* effectively [74]. Moreover, most inactivated vaccines must be administered via parenteral routes, such as subcutaneous or intramuscular routes, and require at least two doses. These requirements significantly increase the labor and economic costs associated with farming operations [75].

#### 3.2.3. Subunit Vaccines

Subunit vaccines present a promising approach to controlling *Salmonella* infections in poultry. Unlike live attenuated or inactivated whole-cell vaccines, subunit vaccines do not contain the entire pathogen and therefore stimulate c immune responses against the specific immunogenic and protective antigenic components included in the vaccine. Thus, subunit vaccines are safer than live or inactivated whole-cell vaccine types as they minimize potential adverse side effects by generating an immune response to targeted antigens only. Examples of antigens used in experimental poultry *Salmonella* subunit vaccines include outer membrane proteins (OMPs), flagella (FLA) antigens, and outer membrane vesicles (OMVs) [76,77]. Li et al. (2020) reported that recombinant OMP F (rOmpF) mixed with QuilA adjuvant and extracted OMVs alone induced strong antibody and cell-mediated immune responses, protecting vaccinated chickens from a subsequent challenge with *S.* Enteritidis and effectively reducing intestinal *Salmonella* colonization [78].

Subunit vaccines have gained special interest due to their safety and specificity in targeting *Salmonella*. Among these, nanoparticle-based vaccines, such as chitosan nanoparticle (CS-NP) formulations, have emerged as a promising approach for immunizing poultry [79]. CS-NPs have been shown to enhance both mucosal and systemic immune responses due to their biocompatibility, biodegradability, and ability to encapsulate antigens. Recent studies have also investigated alternative delivery methods of CS-NP-formulated vaccines, including gel sprays, which optimize vaccine distribution and uptake. For example, Han et al. (2020) demonstrated that gel spray delivery of a CS-NP vaccine encapsulating OMP and FLA antigens, either through drinking water or mixed into feed, resulted in significant mucosal and systemic antibody responses as well as cell-mediated immune response, thereby successfully reducing *Salmonella* colonization in vaccinated chickens. Experiments conducted with different dosages of CS-NP revealed the importance of optimal dosing to balance immune response while minimizing adverse effects [80,81,82,83,84]. However, there is a significant gap in knowledge regarding the application of these vaccines in commercial-layer chickens, as most studies have primarily focused on broiler chickens [85]. Layer chickens represent a distinct group of poultry with unique physiological and production characteristics, which could influence vaccine efficacy and outcomes [86]. Addressing these gaps, including the effects of different delivery routes and dosage regimens in layer chickens, is crucial for developing effective nanoparticle-based vaccines to control *Salmonella* in diverse poultry production systems. Investigating these aspects in future research will greatly enhance the understanding and applicability of nanoparticle-based vaccines, ultimately improving poultry health and food safety.

#### 3.2.4. Ghost Vaccines

Bacterial ghost vaccines represent a novel strategy used in developing poultry *Salmonella* vaccines. These vaccines are prepared from genetically modified Gram-negative bacteria that have had their cytoplasmic contents removed while leaving the cellular membranes intact. This is typically achieved by the controlled expression of the lysis gene E from bacteriophage PhiX174 in bacteria, which triggers the formation of transmembrane channels to release intracellular contents [87,88]. The remaining empty cellular envelopes, so-called “ghosts”, would still maintain the native antigenic structures of bacteria, thereby retaining the capability to induce strong immune responses with a reduced risk of infection [89]. One significant application of this technology is the construction of a ghost vaccine against *Salmonella* Gallinarum to combat fowl typhoid [89]. Chaudhari et al. (2012) reported that the *S. Gallinarum* ghost vaccine was safe and elicited strong antibody- and cell-mediated immune responses, providing protection against *S. Gallinarum* [90]. Recent studies on ghost vaccines have incorporated adjuvants to enhance immunogenicity. For example, Jawale et al. (2014) constructed an *S.* Typhimurium-derived ghost vaccine expressing the heat-labile enterotoxin B subunit (LTB) of *Escherichia coli*, which acted as an adjuvant. Incorporation of LTB significantly enhanced systemic and mucosal antibody responses in vaccinated chickens [91]. When these chickens were subsequently challenged with *S.* Typhimurium, vaccinated chickens exhibited reduced *Salmonella* counts in the internal organs compared to unvaccinated chickens [91]. Similarly, adding surface-displayed FliC as an adjuvant elicited robust antigen-specific immune responses, both humoral and cell-mediated immunity, and significantly reduced bacterial loads in target organs after a challenge with virulent *Salmonella* [92]. Despite the advantages of ghost vaccines—such as increased safety due to the absence of live pathogens and enhanced immunogenicity from the retention of native antigenic structures that provoke a robust immune response— there are still some challenges with regard to their optimization, stability, and consistent immunogenicity across different *Salmonella* serovars [93]. Ongoing research aims to address these challenges and develop improved ghost vaccines that can be integrated into overall *Salmonella* control programs in poultry production.

**Table 1:** Overview of currently used vaccines and their efficacy.

Vaccine Name	Constituents	Outcomes	Routes and Frequency of Administration
**Live attenuated**			
Nobilis^®^ SG 9R, (Merck & Co., Inc., Rahway, NJ, USA)	Live attenuated *S.* Gallinarum (SG) 9R strain with mutated *galE* gene [94]	Reduced *Salmonella* prevalence in vaccinated flocks compared to the unvaccinated control group [95].	Two times via subcutaneous route: 6 weeks and 14–16 weeks of age [95].
Avipro^®^ Megan Vac 1 (Elanco, Greenfield, IN, USA)	*S.* Typhimurium *cya crp* mutant [94]	Reduction in *Salmonella* colonization in ceca and reproductive tracts of vaccinated chickens [33,96]. Reduction in horizontal transfer and liver, spleen, ovary, and cecal colonization of *S.* Enteritidis [97].	Three times via drinking water: 1 day, 2 weeks, and 5 weeks of age [33,96].
Avipro^®^ Megan Egg (Elanco, Greenfield, IN, USA)	*S.* Typhimurium *cya*, *crp* mutant strain χ3985 [66,98]	Reduced *Salmonella* colonization in the ceca, spleen, ovary, and bursa in vaccinated birds [66,98].	Three times via coarse spray: 2, 4, and 16 weeks of age [99].
Vaxsafe^®^ ST, Bioproperties, (Glenorie, Australia)	Attenuated *aroA* deletion *S.* Typhimurium strain STM-1 [100]	Reduced excretion of *Salmonella* in the vaccinated group [100].	Four times: on day 1, via coarse spray; at 2 and 6 weeks of age, via drinking water; and at 12 weeks of age, via intramuscular route [101,102].
Salmovac^®^ SE (Ceva, Libourne, France)	Attenuated *S.* Enteritidis strain 441/014 [103]	Reduced *Salmonella* colonization in ceca and invasion of internal organs [104].	Three times via drinking water: 1, 6, and 13 weeks of age [104].
Gallivac^®^ SE (Merial, France)	*S.* Enteritidis *Ade* and *His* mutant [94]	Reduced colonization of *Salmonella* in cecum and liver [105].	Two times via drinking water: 1 and 15 days of age [105].
Poulvac^®^ ST, Zoetis (Parsippany, NJ, USA)	*aroA* mutant *S.* Typhimurium [106]	A 50% reduction in *S.* Kentucky, *S.* Enteritidis, *S.* Heidelberg, *S.* Typhimurium, and *S.* Hadar recovery from internal organs of vaccinated birds [106].	Two times: on day 1, via coarse spray, and at 2 weeks of age, via drinking water [106].
**Inactivated vaccines**			
Nobilis^®^ Salenvac TMSD animal health, Rahway, NJ, USA	Formalin killed *S.* Enteritidis and *S.* Typhimurium bacterin [107]	Reduction in *Salmonella* shedding and colonization of internal organs (liver and spleen) [70,108].	Two times via intra-muscular route: 1 day and 4 weeks of age [70,108].
Layermune^®^ SE (Ceva Biomune, Lenexa, KS, USA)	Killed *S.* Enteritidis [109]	Reduction in *Salmonella* shedding and colonization of internal organs (liver and spleen) [109].	Two times via subcutaneous route: 5 and 9 weeks of age [109].
Corymune^®^ 4K and 7K (CEVA Corp., Libourne, France)	Killed *S.* Enteritidis [71,109]	Reduction in *Salmonella* shedding and colonization of internal organs (liver and spleen) [109].	Two times via intramuscular route: 5 and 9 weeks of age [109].
Poulvac^®^ SE (Zoetis, Parsippany, NJ, USA)	Formalin killed *S.* Enteritidis, Phage Types 4, 8 and 13a [110]	Reduction in *Salmonella* colonization in ceca, liver, and spleen after challenge on day 1 [72].	Two times via subcutaneous route: 12 and 20 weeks of age [72,111].
AviPro^®^ 109 SE4 Concentrate (Elanco, Greenfield, IN, USA)	Killed *S.* Enteritidis [112]	Reduced colonization of *Salmonella* in internal organs, including reproductive tract.	Two times: first, via subcutaneous route between 12 and 16 weeks of age and booster vaccination 4 weeks later [113].
Avipro^®^ 329 ND-IB2-SE4 Concentrate (Elanco, Greenfield, IN, USA)	Killed chicken bronchitis and Newcastle disease viruses and killed *S.* Enteritidis [114]	Reduction in *S.* Enteritidis colonization in the ceca [114].	Three times: first via subcutaneous route at 12 and 16 weeks of age or intramuscular route at 13 and 17 weeks of age, followed by vaccination with *S.* Enteritidis monovalent vaccine 4 weeks later [99].

### 3.3. Feed Additives

Feed additives can be classified into several main categories, such as probiotics, prebiotics, organic acids, short- and medium-chain fatty acids, essential oils, and bacteriophages, each playing a distinct role in reducing pathogen colonization. It has been reported that these additives modify intestinal microflora to enhance the overall gut health of the bird, which, in turn, helps reduce *Salmonella* colonization.

#### 3.3.1. Probiotics

The Food and Agriculture Organization of the United Nations (FAO)/World Health Organization (WHO) defines probiotics as live microorganisms that, when administered in adequate amounts, confer a health benefit on the host [115]. Probiotics have gained significant attention as a feed additive for controlling *Salmonella* in poultry and have been extensively studied for their ability to outcompete harmful bacteria like *Salmonella* in the GIT [116,117,118]. In addition to competitive exclusion, these probiotics are known to produce antimicrobial compounds and modulate the immune system of the host to interfere with pathogen growth [119,120]. Probiotics have demonstrated significant potential to enhance intestinal development and microarchitecture, which contributes to improved gut health and pathogen resistance [121]. Table 2 shows the mechanism of action of some probiotics used in the poultry industry. Research indicates that probiotic supplementation can lead to increased villus height and reduced crypt depth in the intestinal epithelium, both indicators of enhanced nutrient absorption and improved intestinal integrity [122].

#### 3.3.2. Prebiotics

Prebiotics are defined by the International Scientific Association for Probiotics and Prebiotics (ISAPP) as “a substrate that is selectively utilized by host microorganisms conferring a health benefit” [124]. Specifically, prebiotics serve as selective nutrients for beneficial colonic bacteria, promoting their growth and metabolic activity. Although these compounds are indigestible by the host organism, they provide numerous health benefits through multiple mechanisms. For instance, mannan-oligosaccharides (MOS) and β-glucans have been identified as effective prebiotics that can inhibit the adhesion of *Salmonella* to intestinal epithelial cells, thereby reducing colonization [125]. Currently, probiotics and prebiotics are also used as a combination called synbiotics to exploit their synergistic effects in controlling *Salmonella*, and this combined approach has shown to be more effective than probiotics or prebiotics alone (Table 3). This synergistic combination is intended to enhance both the viability and functionality of probiotics in the gastrointestinal tract while promoting a balanced gut microbiome. Because synbiotics can facilitate the growth of beneficial bacteria, they may offer various health benefits to the host, such as improved digestive health, a strong immune system, and improved general well-being [126].

#### 3.3.3. Postbiotics

Postbiotics are non-viable microbial products or metabolic byproducts from probiotic microorganisms. They have received considerable attention because of their potential ability to reduce *Salmonella* colonization in poultry. Unlike live probiotics, postbiotics consist of short-chain fatty acids, bacteriocins, enzymes, and cell wall fragments, which may offer health benefits without the associated risks of using live bacteria. These traits make them particularly appealing in poultry farming, where preserving intestinal health is crucial for preventing colonization by undesirable bacteria [127,128]. Studies have demonstrated the effectiveness of postbiotics in reducing *Salmonella* colonization in poultry. In this context, a report in Feedinfo by Expana highlighted that specific postbiotic formulations could dramatically reduce *Salmonella* colonization in poultry through modulation of the immune response in the intestines, creating an environment that inhibits the growth of pathogenic bacteria and consequently reducing the likelihood of intestinal colonization in the GIT [129]. Postbiotics such as the *Saccharomyces cerevisiae* fermentation product (SCFP) improve gut health by maintaining immune robustness and enhancing digestive efficiency. This dual action helps control *Salmonella* colonization and improve bird performance, animal welfare, and food safety, particularly benefiting antibiotic-free poultry production systems [130]. One of the advantages of postbiotics over traditional probiotics is their non-viable nature, which reduces problems with the viability and stability of living microorganisms during feed production and storage. Postbiotics also have a lower potential for transferring antibiotic resistance genes, addressing one of the major current issues in animal agriculture [131]. As the poultry industry continues to strive for sustainable and efficient methods of controlling *Salmonella* infections, postbiotics are an attractive non-antibiotic alternative that seems to resonate with consumer and regulatory demands aimed at reducing antibiotic usage.

**Table 3:** Efficacy of prebiotics and synbiotics in controlling *Salmonella*.

Prebiotics	Outcome	Reference
Mannan-rich yeast cell wall-derived preparation	Significant reduction in *Salmonella* recovered from ovaries and up to 1 log unit reduction in *Salmonella* in the ceca and *Salmonella*-challenged birds.	[132]
Fructo-oligosaccharides	Dose-dependent reduction in *S.* Enteritidis in the ceca up to 1.3 log_10_ in orally challenged birds. No change in *Salmonella* isolation from the internal organs (liver, gall bladder, ovary). Increase in Toll-like receptor 4 (TLR 4), interferon-γ (IFN-γ), and IgA expression indicating cell-mediated immune activation.	[133]
**Synbiotics**		
*Bacillus subtilis* and yeast cell wall-derived glucomannan	Reduction in *S.* Enteritidis counts in ceca up to 0.73 log_10_ CFU/g.	[115,116]
*Enterococcus faecium*, *Pediococcus acidilactici*, *Bifidobacterium animalis*, *Lactobacilus reuteri* + Fructo-oligosaccharides	Improvement in vaccine efficacy by reducing *Salmonella* counts in the cecal contents.	
BacPack^®^ Quality Technology International, Inc., Elgin, IL, USA. Combination of a *Bacillus subtilis* strain and *Saccharomyces cerevisiae* cell wall	Reduction in cecal *S.* Enteritidis counts at 11, 15, and 19 days post-challenge.	[134]
*Bacillus subtilis*, *B. licheniformis* + mannooligosaccharide	Reduction in cecal *S.* Enteritidis counts in the ceca and ovaries of challenged birds.	

#### 3.3.4. Organic Acids, Short- and Medium-Chain Fatty Acids

Organic acids, in combination with short-chain and medium-chain fatty acids (SCFAs and MCFAs), are increasingly being used within the poultry-production market as pre-harvest interventions to control *Salmonella* colonization. Such compounds have antimicrobial properties, either inhibiting or reducing the distribution of *Salmonella* in the avian gut. Similarly, organic acids, such as formic and acetic acids, in their undissociated form, can pass through bacterial cell walls and cause intracellular acidification, which results in a disturbance of metabolic functions and, consequently, the inhibition of bacterial growth [135]. SCFAs and MCFAs exhibit membrane-active properties and disrupt bacterial cell membranes, thereby enhancing permeability and leakage of critical cellular components. MCFAs have been especially potent against *Salmonella* [136]. When added to poultry feed, these acids have been associated with a significant reduction in the colonization of *Salmonella*. When combined with added ingredients, such as essential oils or probiotics, they can enhance antimicrobial activity [137]. However, it is important to consider aspects such as maximum dosage, the potential for the development of resistance, and regulatory considerations to ensure their safe and effective use in poultry production. One study demonstrated that diets supplemented with a mix of coated essential oils and organic acids in broilers (a) improved growth performance and gut health and (b) reduced *S.* Enteritidis load in challenged birds [138]. Further, research has shown that medium-chain fatty acids had a stronger antibacterial action on *Salmonella* than short-chain fatty acids, highlighting the importance of selecting the right type of fatty acids for effective control [58,136]. These examples certainly strengthen the prospect of organic acids and fatty acids as potential alternatives to antibiotics for the control of *Salmonella* in poultry.

#### 3.3.5. Essential Oils

Essential oils (EOs) have garnered significant attention as natural antimicrobial agents in poultry production, particularly for their potential to control *Salmonella* colonization at the pre-harvest level. Derived from aromatic plants, EOs such as thyme, oregano, and lemongrass contain active compounds like thymol, carvacrol, and citral, which exhibit potent antimicrobial properties against various pathogens, including *Salmonella*. These compounds disrupt bacterial cell membranes, leading to increased permeability and leakage of essential cellular components, ultimately resulting in bacterial cell death. Additionally, EOs interfere with bacterial enzyme activity and genetic material, hindering bacterial replication and survival [139]. Incorporating EOs into poultry feed or water has been shown to reduce *Salmonella* prevalence in the GIT, thereby enhancing food safety. For instance, a study demonstrated that a combination of essential oils, including eucalyptus, thyme, and lemon, administered in drinking water at a concentration of 0.05% significantly reduced *Salmonella* contamination in the crop of the bird, leading to decreased cross-contamination during slaughter and processing [140]. Another study highlighted that dietary supplementation with a blend of essential oils and organic acids improved growth performance and intestinal health while reducing *S.* Enteritidis load in infected chickens [138]. Although EOs offer a natural alternative to synthetic antimicrobials, several factors must be considered for their effective application in poultry production. Determining optimal concentrations is crucial to ensure efficacy without any adverse effects on bird health or product quality. EOs can be volatile and may degrade during feed processing; therefore, encapsulation techniques are often employed to enhance stability. Additionally, high concentrations may impart unpleasant strong flavors or odors to meat and eggs, potentially affecting consumer acceptance. Therefore, careful formulation and dosage optimization are essential to maximize the benefits of EOs in controlling *Salmonella* in poultry.

#### 3.3.6. Bacteriophages

Bacteriophages are viruses that specifically infect bacteria and are considered non-toxic to humans and animals. Due to their targeted action and safety, bacteriophages have been widely studied as promising alternatives to conventional antimicrobials for pathogen control in various applications [141,142,143,144]. Their host specificity allows them to effectively target pathogenic bacteria while preserving the resident microflora, making them a safer option for pathogen control [145]. Several commercial bacteriophage preparations have been developed to be used against *Salmonella* in poultry. For example, BAFASAL^®^ (Proteon Pharmaceuticals, Lodz, Poland) is a commercial bacteriophage product designed for on-farm administration to poultry during the rearing stage. Studies have demonstrated that BAFASAL^®^ could reduce *Salmonella* levels by up to 200-fold in treated groups compared to untreated groups, with minimal impact on the final product. Importantly, this product requires no withdrawal period for meat or eggs due to its minimal residual effects [146]. Another example is SalmoFREE, a bacteriophage mixture targeting *Salmonella* that has shown complete elimination of *Salmonella* in cloacal swabs of treated poultry. SalmoFREE also appears to confer some residual protection, as subsequent flocks exposed to the environment retained *Salmonella* resistance, likely due to remaining bacteriophage activity [147]. BioTector, produced by Cheil Jedang Corporation in Korea, is another commercial bacteriophage formulation designed to reduce *S. Gallinarum* and *Salmonella* Pullorum in poultry. This product has significantly reduced the prevalence of *Salmonella* in broilers and layers, demonstrating its effectiveness as a mitigation strategy for these pathogens in poultry operations [148]. This suggests that BioTector may also be effective against NTS.

### 3.4. Competitive Exclusion (CE)

The concept of competitive exclusion (CE) in poultry, introduced by E. Nurmi, is based on the idea that the natural gut flora of chickens can inhibit the growth of pathogenic bacteria [149]. Unlike probiotics, which consist of defined bacterial cultures, CE cultures contain a diverse range of microorganisms derived from the gut flora of healthy, mature birds. These CE cultures are intended to be administered to young chicks to reduce their susceptibility to *Salmonella* colonization before establishing their own stable microflora [150]. CE cultures exert pathogen-elimination effects through multiple mechanisms, including competition for attachment sites and nutrients [151], production of antimicrobial substances such as organic acids [152], and modulation of the bird’s immune system [153]. In some cases, even live *Salmonella* vaccines can colonize the intestines of the vaccinated chicks, effectively excluding pathogenic bacteria through competitive interactions. It has been observed that the inhibitory effect is typically stronger within the same serovar than across different serovars of the vaccine [154]. Furthermore, combining CE cultures with live vaccines has shown additive effects in controlling *Salmonella* in vaccinated birds [155]. Several CE culture products have been developed for pathogen control in poultry [156], though only a few, such as Aviguard^®^ (MSD Animal Health, Rahway, NJ, USA) and Broilact^®^ (Orion Pharma Animal Health, Espoo, Finland), are commercially available today. This limited availability may stem from inconsistent results observed during various stages of product evaluation [156,157].

### 3.5. Genetic Approaches

Genetic approaches offer promising strategies for pre-harvest control of *Salmonella* in poultry, thereby improving the intrinsic resistance of avian species to infection. Breeding programs aim to identify and spread genetic traits associated with increased resistance against colonization by *Salmonella*. Studies have revealed that various lines of chickens exhibit varying levels of susceptibility to infection with *Salmonella*, which suggests a genetic component of resistance against the disease [158]. By selecting resistant traits, poultry producers can develop flocks that are less susceptible to *Salmonella* colonization, thereby reducing the risk of contamination in the food supply. Advancements in genomic technologies have facilitated the identification of specific genes and quantitative trait loci (QTL) associated with *Salmonella* resistance. Genome-wide association studies (GWAS) have identified specific genetic loci associated with resistance to *S. Pullorum* in chickens. Notably, a significant region on chromosome 4 has been linked to mortality resulting from *S. Pullorum* infection. Within this region, the single nucleotide polymorphism (SNP) rs314483802 accounts for 11.73% of the observed phenotypic variation in resistance. Candidate genes within this locus, such as FBXW7 and LRBA, were found to downregulate expression following infection, suggesting their roles in mediating resistance [159]. These findings might enable the development of genetic markers that can be used in marker-assisted selection (MAS) programs, allowing for the efficient breeding of resistant poultry lines. Additionally, understanding the genetic basis of immune responses in chickens can inform the design of more effective vaccines and immunotherapies, further bolstering pre-harvest *Salmonella* control measures.

However, implementing genetic approaches in poultry production presents challenges. The genetic diversity within and between poultry populations necessitates comprehensive studies to identify resistance-associated genes across different breeds and environments. Moreover, the potential trade-offs between disease resistance and other economically important traits, such as growth rate and egg production, must be carefully gauged to ensure that overall productivity is not compromised [160]. Despite these challenges, integrating genetic strategies with traditional biosecurity measures and vaccination programs holds significant potential for reducing *Salmonella* prevalence in poultry, thereby enhancing food safety and public health outcomes [161].

### 3.6. Antimicrobial Use

Prophylaxis use of antimicrobials has been a cornerstone in pre-harvest strategies to control *Salmonella* in poultry production for some time. Administering antibiotics to poultry flocks aims to reduce or eliminate *Salmonella* colonization in the GIT, thereby decreasing the risk of contamination during processing. Commonly used antimicrobials include tetracyclines, sulfonamides, and fluoroquinolones, which target a broad spectrum of bacterial pathogens [162]. However, the extensive use of antibiotics in poultry has raised significant concerns regarding AMR development. *Salmonella* strains resistant to multiple antibiotics have emerged, complicating treatment options for human infections and posing a public health risk. The WHO has highlighted the critical importance of addressing AMR in food-producing animals to safeguard human health [163]. In 2006, the European Union implemented a comprehensive ban on the use of antibiotics as growth promoters in animal feed, aiming to combat AMR and safeguard public health [164]. Similarly, the United States has enacted policies to restrict the use of medically important antibiotics in livestock. The US Food and Drug Administration (FDA) has transitioned over-the-counter medically important animal antimicrobial drugs to prescription status, ensuring veterinary oversight and promoting judicious use [165]. These regulatory measures have prompted the poultry industry to seek alternative strategies for *Salmonella* control, such as probiotics, prebiotics, and enhanced biosecurity practices. While antibiotics can effectively reduce *Salmonella* levels in poultry, their use must be judicious and aligned with antimicrobial stewardship principles to mitigate the risk of resistance development. Replacing prophylaxis use of antimicrobials with comprehensive management practices, such as vaccination, biosecurity, and environmental controls, is essential for sustainable *Salmonella* control in poultry production [166].

## 4. Established *Salmonella* Control Programs

National and international *Salmonella* control programs have been established across various countries to address the significant public health risks associated with *Salmonella* contamination in poultry. These programs, spearheaded by government agencies, regulatory bodies, and international organizations, set standards and implement strategies to reduce *Salmonella* prevalence along the poultry production chain. At the international level, agencies such as the WHO, Codex Alimentarius Commission (a joint FAO/WHO program) [167], and the World Organization for Animal Health (WOAH; formerly OIE) [166] provide guidelines and frameworks to support global poultry *Salmonella* control, recognizing that foodborne *Salmonella* infections are a worldwide concern. The EU has also implemented a harmonized *Salmonella* control strategy across its member states, mandating regular testing, biosecurity measures, and vaccination for certain poultry flocks [168,169]. In the United States, the National Poultry Improvement Plan (NPIP) established by the United States Department of Agricultural Sciences (USDA) offers a voluntary yet widely adopted program that sets standards for pathogen monitoring and control within the poultry industry [170]. In addition to the NPIP voluntary program, the FDA fully implemented the mandatory egg safety rule “Prevention of *Salmonella* Enteritidis in Shell Eggs During Production, Storage and Transportation” in 2009 for commercial layer flocks. This rule requires that the flocks with 3000 or more laying hens, whose shell eggs are not processed with treatments like pasteurization, must be tested for *S.* Enteritidis to ensure the safety of table eggs [171,172]. These national and international efforts contribute to reducing the burden of *Salmonella* infections through a unified approach that emphasizes preventive measures, consistent monitoring, and rapid response to contamination incidents. The alignment of programs across borders enhances food safety standards and supports the trade of poultry products, as compliance with recognized control measures is often a prerequisite for international market access.

### 4.1. Testing and Monitoring Programs

Testing and monitoring are core elements of established *Salmonella* control programs. Governments mandate routine sampling and testing for *Salmonella* at different stages of poultry production, including breeder flocks, hatcheries, and layer or broiler operations. For example, the EU’s *Salmonella* Control Program requires member states to test poultry for specific serovars, such as *S.* Enteritidis and *S.* Typhimurium, which are most commonly associated with human illnesses [173]. Testing in these programs is standardized to compare results across farms, regions, and countries. In the United States, USDA’s NPIP sets forth guidelines for testing poultry for *Salmonella*, supported by state and federal oversight [170,174]. Regular testing ensures early detection of *Salmonella*, allowing farms to take immediate corrective actions, such as culling, sanitation, or enhanced biosecurity, to prevent contamination spread. Testing data also contributes to national surveillance systems, informing trends and helping governments assess the effectiveness of control measures [172,175]. The FDA program requires testing the pullet environment for *S.* Enteritidis tested when pullets are 14–16 weeks old and retesting negative flocks at 40–45 weeks. If the environmental test is positive, then eggs (1000 eggs/flock) must be tested for *S.* Enteritidis within 2 weeks of the start of egg laying at 2-week intervals until four consecutive negative tests are obtained. The eggs from positive flocks cannot be sold as shell eggs and must be diverted for pasteurization or another form of treatment [176].

### 4.2. Vaccination Requirements

Vaccination is a preventive strategy that many *Salmonella* control programs incorporate to reduce *Salmonella* colonization in poultry. In the EU, for instance, vaccination of laying hens against *S.* Enteritidis is mandatory [177]. Vaccination policies typically target high-risk poultry populations, such as breeder and layer flocks, as these groups are crucial for preventing vertical transmission of *Salmonella* from parent to offspring. Vaccines in these programs may include live attenuated or inactivated types, each formulated to provide immunity against specific *Salmonella* serovars [62]. Governments and regulatory bodies reduce the risk of *Salmonella* transmission by mandating vaccinations, lowering bacterial loads in birds, and, ultimately, reducing contamination levels in poultry products [177,178]. Vaccination programs are carefully monitored to ensure compliance and effectiveness, with follow-up testing often used to verify immunity levels within flocks. The FDA currently supports voluntary vaccination of commercial layers against *S.* Enteritidis. The agency has noted that while laboratory studies suggest that vaccines can help reduce *S.* Enteritidis colonization in hens and eggs, the field trials have not provided consistent evidence to make vaccination mandatory. Field studies showed that vaccinated flocks often had similar *S.* Enteritidis positive rates as unvaccinated hens, indicating that vaccination alone may not be effective under field conditions. As a result, the FDA views vaccination as an optional supplementary measure to augment the effectiveness of required *S.* Enteritidis control strategies rather than a substitute for them [52].

### 4.3. Biosecurity Protocols

Biosecurity is foundational in preventing *Salmonella* from being introduced and spread within poultry farms. Established biosecurity protocols [172] require farms to control farm access, implement sanitation procedures, and restrict personnel movement between different flock areas. For example, the USDA provides biosecurity guidelines under the NPIP advising farms to use footbaths, sanitize equipment, and implement rodent control measures [179]. The FDA also recommends implementing stringent biosecurity practices to ensure no introduction or transfer of *S.* Enteritidis into or among poultry houses. These measures include protecting against cross-contamination when equipment is moved among poultry houses, preventing cross-contamination when people move between poultry houses, keeping stray poultry, wild birds, cats, and other animals out of poultry houses, and prohibiting employees from keeping birds at home [52]. In Europe, biosecurity standards are similarly rigorous, often forming a key component of each country’s national *Salmonella* control plan under EU legislation. Biosecurity protocols help to control both vertical and horizontal transmission of *Salmonella*, limiting contamination risks from external sources, such as wildlife, equipment, and personnel. Regular government inspections ensure that farms adhere to biosecurity standards, addressing lapses that could increase contamination risks [26].

### 4.4. Certification and Quality Assurance

Certification programs offer poultry producers recognition for compliance with *Salmonella* control standards, boosting consumer confidence and market access. The NPIP provides a voluntary certification program in the United States that indicates pathogen control compliance, including *Salmonella* monitoring [180,181]. Certification programs support producers by validating their biosecurity and hygiene measures, often making their products more marketable domestically and internationally. In the EU, similar certification programs award “*Salmonella*-controlled” or “*Salmonella*-free” status to flocks that meet specific pathogen reduction criteria. Certified producers benefit from consumer trust; certain markets may require these certifications as part of import regulations. Certification programs reinforce adherence to control standards and incentivize producers to maintain best practices in *Salmonella* management [173,182].

### 4.5. Surveillance and Reporting Systems

Surveillance and reporting systems track *Salmonella* prevalence in poultry and human populations, providing insights into outbreak patterns, high-risk serovars, and the effectiveness of control measures. These systems integrate data from routine testing, farm inspections, and human health surveillance, offering a comprehensive view of *Salmonella* trends. The European Food Safety Authority (EFSA) consolidates *Salmonella* data from EU member states, monitoring changes in prevalence and identifying emerging risks [183]. In the United States, the Centers for Disease Control and Prevention (CDC) collaborates with the USDA and other agencies to monitor *Salmonella* trends through PulseNet and FoodNet national laboratory network systems that detect and track foodborne outbreaks [184,185]. Surveillance systems enable rapid response to outbreaks, allowing government agencies to trace contamination sources and adjust policies to address identified weaknesses in the supply chain.

### 4.6. Research and Development Initiatives

Research and development (R&D) are essential for established *Salmonella* control programs, as they help evolve and improve existing control strategies. Many programs invest in R&D to develop new vaccines, enhance biosecurity technologies, and explore antibiotic alternatives. The USDA, for instance, funds research focused on alternative pathogen control methods, which aim to reduce *Salmonella* prevalence in poultry without relying on antibiotics. Similarly, the EFSA supports research to better understand *Salmonella* epidemiology in poultry, identifying high-risk transmission points and exploring innovative control solutions. These research efforts provide evidence-based guidance for refining control programs, addressing challenges such as AMR, and adapting to evolving *Salmonella* serovar profiles. Investment in R&D strengthens *Salmonella* control programs, ensuring that they remain effective in changing agricultural and public health landscapes [183,186]

### 4.7. Farmer Education and Outreach Programs

Education and outreach are crucial for the successful implementation of *Salmonella* control measures. Government agencies and industry bodies conduct training programs and workshops, providing resources to inform farmers about *Salmonella* risks, biosecurity practices, and hygiene protocols. Education programs often cover areas such as handling procedures, equipment sanitation, pest control, and proper waste disposal. For example, the USDA collaborates with poultry industry associations to conduct biosecurity training for farm staff, while the EU funds awareness campaigns to help producers comply with *Salmonella* control regulations. Educating producers and farm workers on best practices enables them to implement effective control measures, reducing *Salmonella* risks at the farm level. Outreach programs promote consistency across farms of all sizes, enhancing overall program compliance and food safety outcomes [179,187].

### 4.8. Implementation of Alternative Pathogen Control Methods

Some control programs have begun to adopt alternative pathogen control methods to reduce reliance on antibiotics, thereby addressing AMR concerns. These alternatives include the use of probiotics, prebiotics, synbiotics, postbiotics, and organic acids, which help maintain a healthy gut microbiome in poultry and reduce *Salmonella* colonization. Programs may provide guidelines on approved products, including their usage and dosage, to ensure safe and effective application. For instance, in certain European countries, government guidelines recommend that organic acids be added to feed and water as a preventive measure against *Salmonella*. Similarly, the USDA supports research and industry adoption of probiotic use to manage gut health in poultry [188,189]. By promoting alternatives to antibiotics, *Salmonella* control programs help mitigate AMR and support sustainable poultry production.

## 5. Challenges and Limitations in Pre-Harvest Control Measures

Controlling NTS in poultry during the pre-harvest phase presents several challenges and limitations. One significant issue is the asymptomatic carriage and intermittent shedding of *Salmonella* in poultry, allowing the bacteria to persist undetected within flocks and complicating early identification and intervention efforts [190]. Environmental factors, such as contaminated feed, water, and litter, serve as reservoirs for *Salmonella*, facilitating its introduction and spread within poultry operations. Implementing stringent biosecurity measures is essential but can be resource-intensive and challenging to maintain consistently across diverse farming systems. Additionally, while vaccination programs can reduce *Salmonella* prevalence, their effectiveness varies depending on the serovars present and the specific vaccines used [191]. The emergence of antibiotic-resistant *Salmonella* strains further complicates control efforts, as it limits the efficacy of antimicrobial treatments and necessitates the development of alternative strategies. Moreover, the complex interactions between *Salmonella* and the poultry gut microbiome can influence colonization dynamics, making it difficult to predict and manage infection patterns effectively. These challenges underscore the need for an integrated, multifaceted approach to pre-harvest *Salmonella* control in poultry, combining biosecurity, vaccination, environmental management, and ongoing surveillance to effectively mitigate the risk of contamination. While pre-harvest interventions such as probiotics are widely studied for controlling *Salmonella* colonization in poultry, their effects could be transient. Research indicates that probiotic treatments may only reduce *Salmonella* colonization for short periods, sometimes lasting as briefly as one week [192]. Another considerable challenge with probiotic supplements is ensuring the accuracy of the bacterial composition in the final product. This uncertainty creates regulatory hurdles and raises concerns about these formulations’ reliability and effectiveness [193]. Since probiotics are live bacterial cultures, incorporating them into the diets of chickens presents unique challenges. These include preventing probiotic degradation during pelleting while also ensuring shelf stability and cost-effectiveness [194].

## 6. Future Directions and Innovations

Advancements in pre-harvest control of NTS in poultry have focused on new ways to enhance food safety. A very promising area includes the development of bacterio-phage-based interventions, using viruses that specifically target *Salmonella* bacteria and destroy them. These phage therapies may offer a natural and precise way of reducing *Salmonella* colonization in poultry flocks. Additionally, research is exploring the use of prebiotics and synbiotics to modulate the gut microbiota, thereby creating an environment less conducive to *Salmonella* colonization. Genomic technologies are also being employed to identify genetic markers associated with *Salmonella* resistance, facilitating selective breeding programs aimed at developing poultry lines with enhanced resistance to NTS. Furthermore, new developments in rapid diagnostic tools now allow for better detection and monitoring of *Salmonella* at the farm level, enabling timely interventions. These innovations may represent a complete approach to mitigating *Salmonella* risks in poultry production, when applied with traditional biosecurity measures.

In parallel, advanced vaccine preparation and delivery methodologies, such as cochleate-based delivery systems, are being utilized to enhance the immunogenicity of subunit vaccines. Cochleates are specialized structures characterized by their unique spiral morphology composed of solid lipid bilayers. Studies have demonstrated that cochleate formulations elicit enhanced systemic and mucosal immune responses, thereby improving vaccine efficacy [183]. Integrating these advanced vaccine technologies with existing control measures holds promise for more effective control of NTS in poultry.

## 7. Conclusions

Controlling NTS presents an important aspect of safeguarding public health and ensuring sustainability in the table egg industry and other poultry sectors. This requires an integrated approach that includes vaccination, biosecurity measures, environmental management, and rigorous *Salmonella* surveillance and monitoring, as expounded here. This will reduce the prevalence of *Salmonella* in chickens and help control AMR, ensuring the safety of poultry products intended for human consumption. The near future appears promising with the development and introduction of new strategies, such as bacteriophage-based intervention, advances in genomics and poultry breeding, and the introduction of improved vaccines and vaccine delivery techniques. These innovations are expected to create more resilient and efficient *Salmonella* control measures. Collaboration among researchers, policymakers, and industry stakeholders will mutually benefit the poultry industry, public health, and consumers worldwide by improving animal health and economic sustainability and promoting consumer confidence in poultry products.

## Figures and Tables

**Table 2:** Common probiotics in poultry.

Probiotic	Outcome	Reference
*B. subtilis* CSL2	Re-establishment of normal gut flora abundance (phylum Firmicutes and Proteobacteria and genus *Lactobacillus*) that is disrupted after *Salmonella* infection.	[123]
Poultry Star^®^ *Enterococcus faecium*, *Pediococcus acidilactici*, *Bifidobacterium animalis*, and *Lactobacillus reuteri*	Increased the efficacy of the live attenuated vaccine (*aroA* mutant *S.* Typhimurium) and reduced the cecal colonization of *Salmonella.*	[116]
*Bacillus subtilis* DSM 32324, *Bacillus subtilis* DSM 32325, and *Bacillus amyloliquefaciens*	Reduction in *Salmonella* in cecal contents and establishment of normal gut flora after *Salmonella* challenge.	[117]
*Bacillus* *amyloliquefaciens*, *B.* *licheniformis*, and *B.* *pumilus*	Significant reduction in *Salmonella* in cecal contents 7 days after challenge.	[118]

## Data Availability

No additional data is available.
